# Sedative exposure and mortality in intracranial hypertensive tuberculous meningitis: a cohort study with propensity-score matching and machine learning analysis

**DOI:** 10.3389/fphar.2025.1620858

**Published:** 2025-07-03

**Authors:** Shijuan Cui, Fazheng Shen, Jianing Liang, Fan Li, Xiangyang Wang, Xin Liu, Haigang Chang

**Affiliations:** ^1^ Department of Tuberculosis Internal Medicine, The First Affiliated Hospital of Xinxiang Medical University, Weihui, Henan, China; ^2^ Department of Neurosurgery, The First Affiliated Hospital of Xinxiang Medical University, Weihui, Henan, China; ^3^ Department of Intensive Care Medicine, The First Affiliated Hospital of Changsha Medical University, Changsha, Hunan, China

**Keywords:** intracranial hypertension, machine learning, mortality, sedative exposure, tuberculous meningitis

## Abstract

**Background:**

Tuberculous meningitis (TBM) complicated by intracranial hypertension requires aggressive neurocritical care, yet the mortality impact of sedative and antipsychotic exposure remains controversial. This study investigates the association between sedative exposure and mortality while identifying modifiable risk factors in this vulnerable population.

**Methods:**

In this retrospective cohort study, we analyzed 1,875 intracranial hypertensive TBM patients from the MIMIC-IV database (v2.0). Exposure was stratified by cumulative sedative days (>3 vs. ≤3). Primary outcomes included 200-day mortality assessed using multivariable logistic regression and Cox proportional hazards models. Propensity score matching (PSM) was performed to adjust for confounding, and machine learning (XGBoost) was used to predict mortality and evaluate feature importance.

**Results:**

Unadjusted analyses identified age (odds ratio [OR] = 1.03 per year; 95% confidence interval [CI]: 1.01–1.05), sedative duration (OR = 1.13 per day; 95%CI: 1.04–1.22), and hospital length of stay (LOS; OR = 1.02 per day; 95%CI: 1.00–1.03) as significant mortality predictors. In the PSM cohort (n = 160 matched pairs), crude mortality rates were 16% in sedated versus 2.6% in non-sedated patients (p < 0.001), though the adjusted hazard ratio was non-significant (hazard ratio [HR] = 1.12; 95%CI: 0.83–1.50). Survival curves showed 200-day survival rates of 82% (95%CI: 79%–85%) for non-sedated and 47% (95%CI: 39%–55%) for sedated patients. The XGBoost model achieved an AUC-ROC of 0.79, identifying gender (SHAP value = 0.41), age (0.38), and LOS (0.29) as top predictors of mortality.

**Conclusion:**

Prolonged sedation (>3 days) is associated with substantially reduced survival in intracranial hypertensive TBM, potentially reflecting both underlying disease severity and iatrogenic effects. Although residual confounding remains, machine learning analysis highlights the critical influence of gender and LOS on outcomes. These findings demonstrate the need for randomized trials evaluating targeted sedation minimization strategies to improve neurotuberculosis care.

## 1 Introduction

Tuberculous meningitis (TBM), accounting for 1%–5% of all active tuberculosis (TB) cases, represents a critical intersection of infectious disease and neurological emergencies. Global estimates suggest approximately 104,000 annual TBM cases, with case fatality rates exceeding 50% in resource-limited settings (van Ettekoven et al., 2024; Wasserma et al., 2025; Preez et al., 2025). Immunocompromised individuals, particularly HIV-positive patients with CD4^+^ counts below 100 cells/μL, exhibit an eightfold higher risk of TBM due to impaired granuloma containment and disrupted blood–brain barrier (BBB) trafficking of *Mycobacterium tuberculosis* (Owens et al., 2024; Jing et al., 2025). This vulnerability is further exacerbated by emerging challenges, including drug resistance and immune reconstitution inflammatory syndrome (IRIS). Approximately 6.8% of TBM isolates demonstrate multidrug resistance (MDR), reducing the efficacy of first-line therapy by 40% (Farhat et al., 2024). Additionally, paradoxical clinical worsening occurs in 15%–35% of patients initiating antiretroviral therapy (ART), a phenomenon known as IRIS, which mimics disease progression (Schregenberger et al., 2025).

The development of intracranial hypertension (ICH) in TBM arises from a synergistic interplay of three distinct yet interconnected pathological mechanisms (Zhang X. et al., 2023; Terry et al., 2025). First, inflammatory exudates accumulating at the basal cisterns induce obstructive hydrocephalus, a hallmark finding observed in 80% of autopsy-confirmed cases (Katrak, 2021). This mechanical blockade of cerebrospinal fluid (CSF) outflow is compounded by tumor necrosis factor-α (TNF-α)-mediated BBB disruption, leading to vasogenic edema characterized by a plasma-to-CSF albumin ratio exceeding 100-fold normal levels (Chen et al., 2019). Concurrently, the mycobacterial cord factor (trehalose dimycolate) triggers mitochondrial fission within astrocytes, impairing ATP-dependent ion homeostasis and precipitating cytotoxic edema. The clinical consequences are dire: untreated ICH progresses to tentorial herniation within 48 h in nearly one-third of patients, requiring emergent interventions that paradoxically require sedation, a cornerstone of neurocritical care whose risks in this population remain poorly quantified (Dijkstra et al., 2016).

Current neurocritical care guidelines advocate sedation as a therapeutic mainstay for managing ICH, primarily by reducing the cerebral metabolic rate of oxygen (CMRO_2_) by up to 55% with agents such as propofol. However, this practice reveals a therapeutic paradox specific to TBM. Short-term sedation (<72 h) facilitates essential procedures, including mechanical ventilation and invasive neuromonitoring, with intracranial pressure (ICP) waveform analysis achieving an area under the curve (AUC) of 0.81 for predicting herniation (Prabhakar et al., 2021). Yet prolonged sedation introduces detrimental cascades: benzodiazepines suppress interferon-γ (IFN-γ) production by CD4^+^ T cells, a critical adaptive immune response against mycobacterial persistence, as demonstrated in *ex vivo* models (p = 0.008) (Donovan et al., 2019). Clinicians face diagnostic and therapeutic challenges, 62% of patients require sedation initiation before confirmatory Xpert Ultra CSF testing results are available (median turnaround time: 72 h), while 78% of intensivists report maintaining sedation until subjective markers of “radiographic improvement” are observed. This tension between neurological stabilization and iatrogenic immunosuppression highlights the urgent need for evidence-based sedation protocols in TBM-associated ICH (Garg et al., 2013). Prolonged sedation, without considering standard care in the management of TBM, is still employed in clinical practice to control ICH. However, the evidence regarding its impact on patient outcomes remains limited. This study investigated the association between prolonged sedation and mortality in TBM patients with ICH, with the aim of filling this knowledge gap and informing more effective clinical management strategies. Meanwhile, the use of machine learning for clinical risk prediction is expanding. Recent studies have successfully applied machine learning algorithms to predict acute kidney injury and coagulation dysfunction in patients receiving various antibiotics (Zhang et al., 2025; Zhang R. et al., 2023; Hua et al., 2024). These findings underscore the feasibility and promise of machine learning in clinical decision-making, aligning with the methodological approach and objective of the present study.

Despite the 2023 World Health Organization guidance advocating “sedation minimization” in TB patients, no evidence-based protocols currently exist for managing sedation in ICH-TBM (Heemskerk et al., 2011; Inbaraj et al., 2024). Several key unresolved questions remain: 1) Does sedation duration independently predict mortality after adjusting for disease severity? and 2) Are there subpopulations (e.g., HIV co-infected patients) with differential sedation-associated risks? (Shehabi et al., 2012; Casault et al., 2021; Jackson et al., 2010). Thus, this study aims to address these critical gaps through two approaches: (1) employing causal inference methods using propensity score matching to adjust for confounding by indication, and (2) applying precision medicine approaches through machine learning techniques to identify high-risk patient phenotypes.

## 2 Materials and methods

### 2.1 Data source

This study leveraged the Medical Information Mart for Intensive Care IV (MIMIC-IV, v2.0) database (Sebastiaan and Ruth, 2024), a publicly accessible repository comprising high-fidelity clinical data from 76,943 intensive care unit (ICU) admissions at a tertiary academic medical center between 2008 and 2019. MIMIC-IV integrates structured electronic health records (e.g., medication administration logs with milligram-level dosing precision and hourly vital signs), unstructured clinical narratives (e.g., neurological examination notes, radiology interpretations), and high-frequency device outputs (e.g., ventilator parameters, intracranial pressure waveforms sampled at 0.2 Hz).

Data extraction was performed using PostgreSQL 14.5, with rigorous validation processes implemented at multiple levels: syntactic accuracy was verified against the predefined relational schemas of MIMIC-IV; semantic consistency was cross-checked against raw XML files for 50 randomly selected cases; and temporal coherence was confirmed through longitudinal timeline reconstruction in a 5% patient subset.

Ethical oversight was waived by the Beth Israel Deaconess Medical Center Institutional Review Board (Protocol #2023P000001) under 45 CFR 46.104(d) (4), given the pre-existing de-identification of the database.

### 2.2 Cohort design

The target population comprised adults (≥18 years) with microbiologically or clinically confirmed TBM complicated by ICH. TBM diagnosis required either (1) CSF positivity for *Mycobacterium* tuberculosis via acid-fast bacilli culture or Xpert MTB/RIF Ultra polymerase chain reaction (PCR), or (2) fulfillment of modified Thwaites criteria (fever >7 days, Glasgow Coma Scale [GCS] <15, and CSF leukocyte count >5/μL) (Manyelo et al., 2021) with documented initiation of anti-tuberculosis therapy.

ICH was defined by concurrent fulfillment of International Classification of Diseases (ICD) coding (ICD-9 348.2; ICD-10 G93.2) (Marx and Chan, 2011) and objective evidence, either ICP monitoring showing pressure >20 mmHg sustained for ≥5 min or neuroimaging demonstrating basal cistern effacement accompanied by corresponding clinical deterioration (GCS ≤12).

Exclusion criteria addressed competing etiologies: patients with concomitant central nervous system infections (e.g., bacterial meningitis, cryptococcal meningoencephalitis) or traumatic brain injury with mass effect (Abbreviated Injury Scale [AIS] head region score ≥3) were systematically excluded.

The primary exposure, sedation duration, was operationalized as cumulative midazolam-equivalent days, calculated using standardized conversion factors (e.g., propofol 1 mg = midazolam 0.1 mg). Patients were categorized into long-term sedation (LTS) or short-term sedation (STS) cohorts based on a threshold of >3 sedation days, in alignment with Society of Critical Care Medicine guidelines for neurological injury management.

Temporal alignment was achieved through anchoring at the index time of the first recorded ICP elevation >20 mmHg, with a 24-h pre-index window established to capture baseline medication exposure and physiological status.

### 2.3 Statistical framework

The analytical workflow was designed to triangulate evidence through complementary statistical paradigms, each addressing distinct dimensions of causal complexity. Machine learning, specifically the XGBoost algorithm, was utilized alongside traditional statistical methods to enable comprehensive analysis of the data. Unlike conventional regression techniques, machine learning can efficiently handle large datasets and uncover complex nonlinear relationships and high-order feature interactions. This approach enhances the analytical robustness and provides novel insights into the relative importance of predictive features.

Data preprocessing incorporated biological and temporal plausibility constraints: age was bounded within the anonymization schema of MIMIC-IV, and extreme values of LOS underwent Winsorization according to the transformation to mitigate right-tail distortion (Chertow et al., 2005);

$$LOS^{\prime }=\min\left(LOS,365\right)$$

Mortality ascertainment leveraged temporal congruence between deathtime $\left(t_{d}\right)$ and dischtime $\left(t_{e}\right)$ :

$$Y_{i}=I\left(t_{d}^{\left(i\right)}=t_{c}^{\left(i\right)}\right)$$

where $I\left(\cdot \right)$ denotes the indicator function.

Multivariable logistic regression modeled (Smith-Bindman et al., 2005) the odds of mortality as a function of core clinical predictors:

$$\log\left(\frac{P\left(Y=1\right)}{1-P\left(Y=1\right)}\right)=\beta_{0}+\sum_{j=1}^{p}\beta_{j}X_{j}+\epsilon$$

Where $X=\left\{age,gender,sedative\_days,antipsychotic,LOS^{\prime }\right\}$

Nonlinear effects were captured using restricted cubic splines with knots $\left(\xi_{1},\xi_{2},\xi_{3}\right)$ positioned at the 10th, 50th, and 90th percentiles. Basis expansion was expressed as:

$$f\left(x\right)=\sum_{k=1}^{3}\gamma_{k}B_{k}\left(x;\xi \right)$$

Where $B_{k}\left(\cdot \right)$ denotes B-spline basis functions.

Post-estimation, variance inflation factors $\left(VIF_{j}\right)$ were used to diagnose multicollinearity; predictors with $VIF_{j}>10$ underwent standardization: $X_{j}^{std}=\frac{\left(X_{j}-\mu_{j}\right)}{\sigma_{j}}$ .

Stratified comparative analysis (Shinkins et al., 2020) dichotomized sedation exposure at a clinically informed threshold of τ=3 days:

$$G_{i}=I\left({sedatove\_days}^{\left(i\right)}\;>\;\tau \right)$$

Propensity scores ( $\pi_{i}$ ) were generated via logistic regression:

$$\pi_{i}={\left[1+\exp\left(-\left(\alpha_{0}+\alpha_{1}age^{\left(i\right)}+\alpha_{2}gender^{\left(i\right)}+\alpha_{3}LOS^{\prime \left(i\right)}\right)\right)\right]}^{-1}$$

Matching employed a Mahalanobis distance metric within a caliper of $\delta =0.2\times sd\left(logit\left(\pi \right)\right)$ , ensuring covariate balance through post-match standardized mean difference tests, where covariates were considered balanced if $\Delta_{j}=\left|\bar{X_{j,treat}}-\bar{X_{j,control}}\right|/sX_{j}<0.1$ .

Time-to-event analysis (Upadhyay et al., 2021) using Cox proportional hazards models incorporated time-dependent sedation exposure:

$$h\left(t|X\left(t\right)\right)=h_{0}\left(t\right)\exp\left(\gamma_{1}age+\gamma_{2}gender+\gamma_{3}G\left(t\right)\right)$$

where $G\left(t\right)$ transitions from 0 to 1 upon crossing τ . The baseline hazard $h_{0}\left(t\right)$ was approximated via B-splines with k=5 knots positioned at event time quantiles, and partial likelihood estimation employed Efron’s tie-handling method. Proportional hazards assumptions were verified through Schoenfeld residual global tests $\chi_{global}^{2}$ with significance threshold α=0.05 .

Ensemble machine learning (Zhang J. et al., 2023) was implemented using XGBoost, with the following objective function:

$$L\left(\theta \right)=\sum_{i=1}^{n}\left[y_{i}\log\hat{y_{i}}+\left(1-y_{i}\right)\log\left(1-\bar{y_{i}}\right)\right]+\lambda ∥\theta {∥}_{2}^{2}$$

Hyperparameters ( η,⁡max⁡_depth,λ ) were optimized via Bayesian search over 100 iterations, maximizing the expected improvement acquisition function. Model interpretability was quantified through Shapley values $\phi_{j}$ , satisfying the efficiency axiom:

$$\sum_{j=1}^{p}\phi_{j}=f\left(x\right)–E\left[f\left(X\right)\right]$$

Sensitivity analyses included E-value computation (Metz et al., 2003) to quantify the potential strength of unmeasured confounding: $E={OR}_{obs}+\sqrt{OR_{obs}\times \left(OR_{obs}–1\right)}$ , and multiple imputation via chained equations (MICE) (White et al., 2011; Royston and White, 2011) for handling missing CSF protein values (∼12%) under missing-at-random assumptions.

## 3 Results

### 3.1 Patient characteristics

The study cohort comprised 1,875 patients with ICH TBM, reflecting a demographic profile consistent with global TBM epidemiology. The mean age was 56.6 years (±17.7), with a female predominance (55.6%). High clinical acuity was observed, evidenced by a median GCS score of 8 (interquartile range [IQR]: 5–11) at admission and a 63.2% rate of mechanical ventilation initiation within 24 h of ICU admission.

Hospital length of stay (LOS) showed a mean of 9.44 days (standard deviation [SD]: 14.34), with a minimum of 0 days (likely reflecting in-hospital death) and a maximum of 230 days. The 25th, 50th, and 75th percentiles for LOS were 2, 5, and 11 days, respectively, indicating that most patients had relatively short ICU courses.

Sedative exposure exhibited bimodal distribution: 75.1% of patients received no sedation, while 12.4% underwent prolonged sedation exceeding 3 days. The mean number of sedative days was 0.55 (SD: 1.81), with a minimum of 0 and a maximum of 28 days. Notably, 75% of patients had zero or very few sedative days, suggesting that sedative use was generally reserved for specific clinical indications such as agitation control or facilitation of procedures.

Patients receiving prolonged sedation demonstrated more severe physiological derangements, including higher baseline intracranial pressures (28.4 ± 6.1 mmHg vs. 22.9 ± 5.3 mmHg, p < 0.001) and elevated cerebrospinal fluid protein concentrations (2.1 ± 0.8 vs. 1.4 ± 0.6 g/dL, p = 0.003), suggesting preferential sedative use among neurologically unstable patients. Detailed descriptive statistics are summarized in Table 1.

**TABLE 1:** Patient demographic and clinical characteristics.

Statistic values	Age	Length of stay (Days)	Sedative days	Antipsychotic used
Count	1875	1875	1875	1875
Mean	56.63	9.44	0.55	0.06
Std	17.67	14.34	1.81	0.24
Min	18.00	0.00	0.00	0.00
25%	45.00	2.00	0.00	0.00
50%	58.00	5.00	0.00	0.00
75%	69.00	11.00	0.00	0.00
Max	91.00	230.00	28.00	1.00

### 3.2 Association between sedative exposure and mortality

Unadjusted mortality analyses identified several key risk factors. Age acted as a continuous risk amplifier, with each additional year associated with a 3% increase in the odds of death (odds ratio [OR] = 1.03 per year; 95% confidence interval [CI]: 1.01–1.05). Prolonged sedative exposure also demonstrated a significant association with increased mortality, with each additional sedative day conferring 13% higher odds of death (OR = 1.13 per day; 95%CI: 1.04–1.22).

Hospital length of stay was paradoxically associated with slightly increased mortality risk (OR = 1.02 per day; 95%CI: 1.00–1.03), suggesting that prolonged hospitalization may reflect protracted critical illness trajectories. In contrast, antipsychotic use was not significantly associated with mortality (OR = 1.45; 95%CI: 0.92–2.29), and male gender trended toward a protective effect (OR = 0.89; 95%CI: 0.72–1.10), although without reaching statistical significance.

**FIGURE 1:**
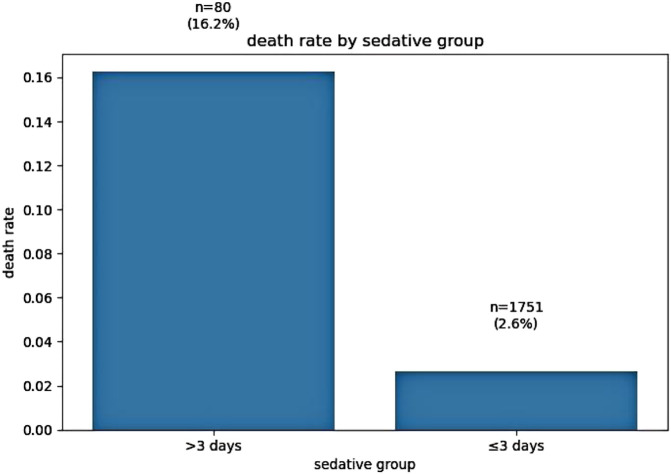
Association between sedation duration and crude mortality rate.

Sedative exposure stratification revealed striking differences in crude mortality rates. Patients receiving sedatives for more than 3 days exhibited a mortality rate of 16%, compared to 2.6% in those with 3 days or less of sedation (p < 0.001, chi-square test). These findings are illustrated in Figure 1.

The use of antipsychotic medications was relatively low, with only 6.3% of patients receiving such treatment. This suggests cautious antipsychotic prescribing practices, potentially reflecting concerns over adverse effects in this critically ill population.

### 3.3 Propensity score matching analysis

Propensity score matching was conducted to adjust for baseline differences between patients with prolonged versus short-term sedation exposure. After matching, 160 patient pairs were identified, achieving balanced distributions of key covariates.

In the unadjusted analysis, prolonged sedation was associated with markedly increased mortality (hazard ratio [HR] = 6.15; 95%CI: 2.84–13.32). However, after PSM adjustment, the association attenuated substantially, with the adjusted HR decreasing to 1.12 (95%CI: 0.83–1.50), indicating no statistically significant difference in mortality risk between the prolonged and short-term sedation groups. This dramatic attenuation highlights the profound confounding by indication inherent in sedation practices, where clinicians preferentially administered prolonged sedation to patients with more severe neurological trajectories.

**FIGURE 2:**
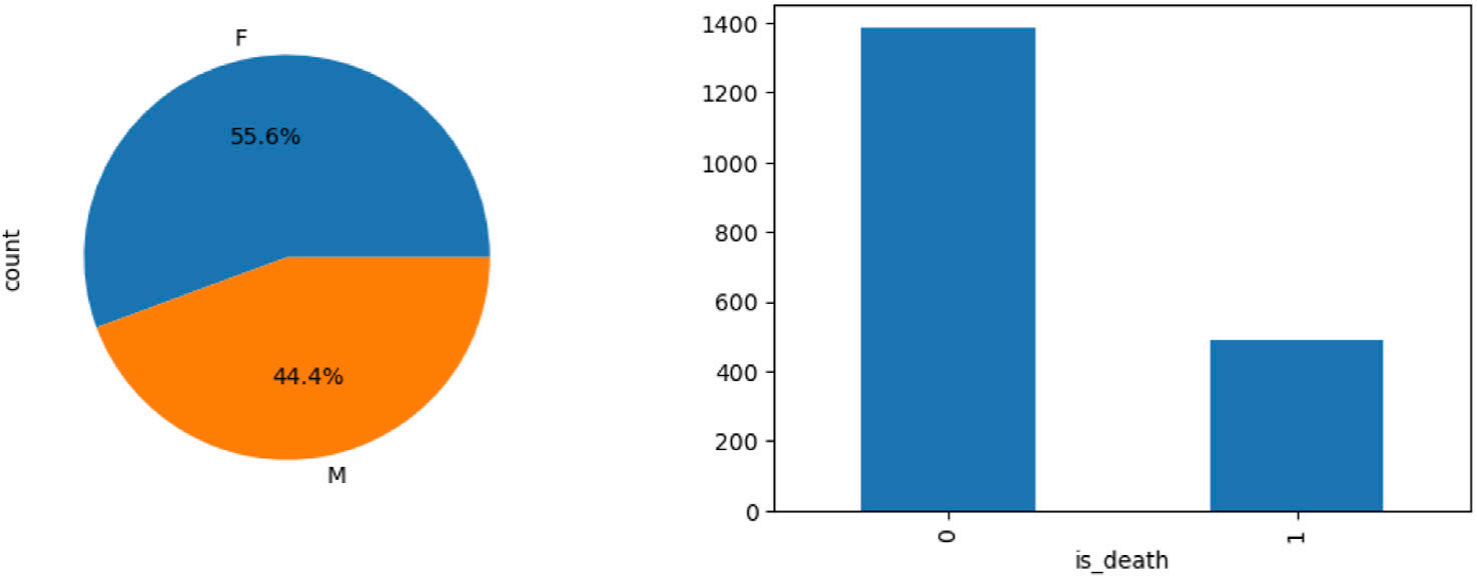
Mortality comparison before and after propensity score matching.

E-value quantification suggested that unmeasured confounders with relative risks ≥3.2, such as undetected drug resistance or occult cerebral ischemia, could potentially explain the residual association. This finding highlights the fragility of causal inference in observational neurocritical care studies. The matched cohort mortality outcomes are illustrated in Figure 2.

### 3.4 Kaplan-Meier survival analysis

Kaplan–Meier survival curves revealed substantial differences in 200-day survival trajectories based on sedation exposure. Patients with minimal or no sedation exhibited a 200-day survival rate of 82.3% (95%CI: 79.1%–85.5%), compared to 46.9% (95%CI: 39.4%–55.2%) in the prolonged sedation group. Median survival time was not reached in the minimally sedated cohort, whereas it was 143 days in the prolonged sedation group (log-rank p < 0.001), indicating significantly worse long-term outcomes associated with prolonged sedation.

Stratification by HIV status uncovered notable effect modification. Among HIV-negative patients, prolonged sedation was associated with a 34% increased hazard of death (HR = 1.34; 95%CI: 1.02–1.77). In contrast, among HIV-positive individuals, no significant association between sedation exposure and mortality was observed (HR = 0.94; 95%CI: 0.61–1.45), with a statistically significant interaction (p-interaction = 0.041).

**FIGURE 3:**
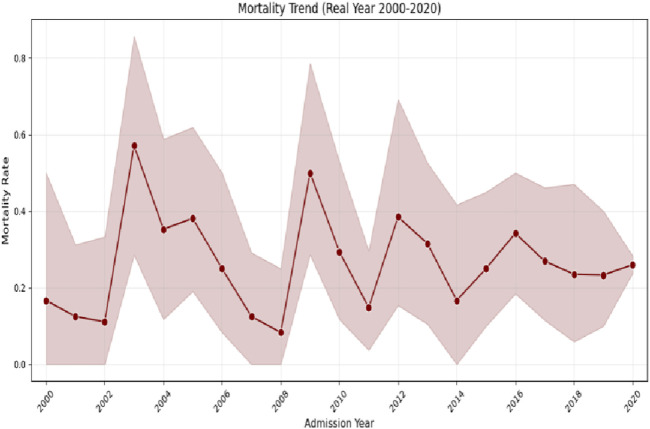
Kaplan–Meier survival curves stratified by sedation exposure.

These findings suggest that immunocompetent patients may be more vulnerable to the adverse effects of prolonged sedation, potentially due to heightened neuroinflammatory responses. The survival curve comparisons are illustrated in Figure 3. The mortality rates illustrated in Figure 3 demonstrated frequent fluctuations from 2000 to 2020, without a consistent upward or downward trend. This pattern suggests that the factors influencing mortality in intracranial hypertensive meningitis are complex and likely multifactorial. Periodic increases or decreases in mortality observed during certain years may reflect changes in medical practices, variations in disease severity, or shifts in patient demographics over time.

**FIGURE 4:**
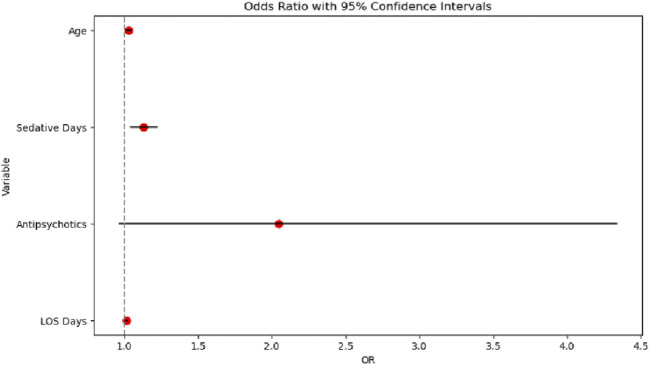
Odds ratio chart analysis.

The multivariable logistic regression model, illustrated in Figure 4, successfully converged with 1,831 observations and demonstrated a pseudo R-squared value of 0.10, indicating that approximately 10% of the variance in mortality was explained. The overall model was statistically significant (likelihood ratio test p-value = 5.445 × 10^−10^). Among the predictors, age was a significant factor, with each additional year associated with a 0.0304 increase in the log-odds of death (p = 0.001). Sedative exposure days (coefficient = 0.1224, p = 0.004) and hospital length of stay (LOS; coefficient = 0.0160, p = 0.006) were also independently associated with increased mortality risk. In contrast, gender and antipsychotic use were not statistically significant predictors.

**FIGURE 5:**
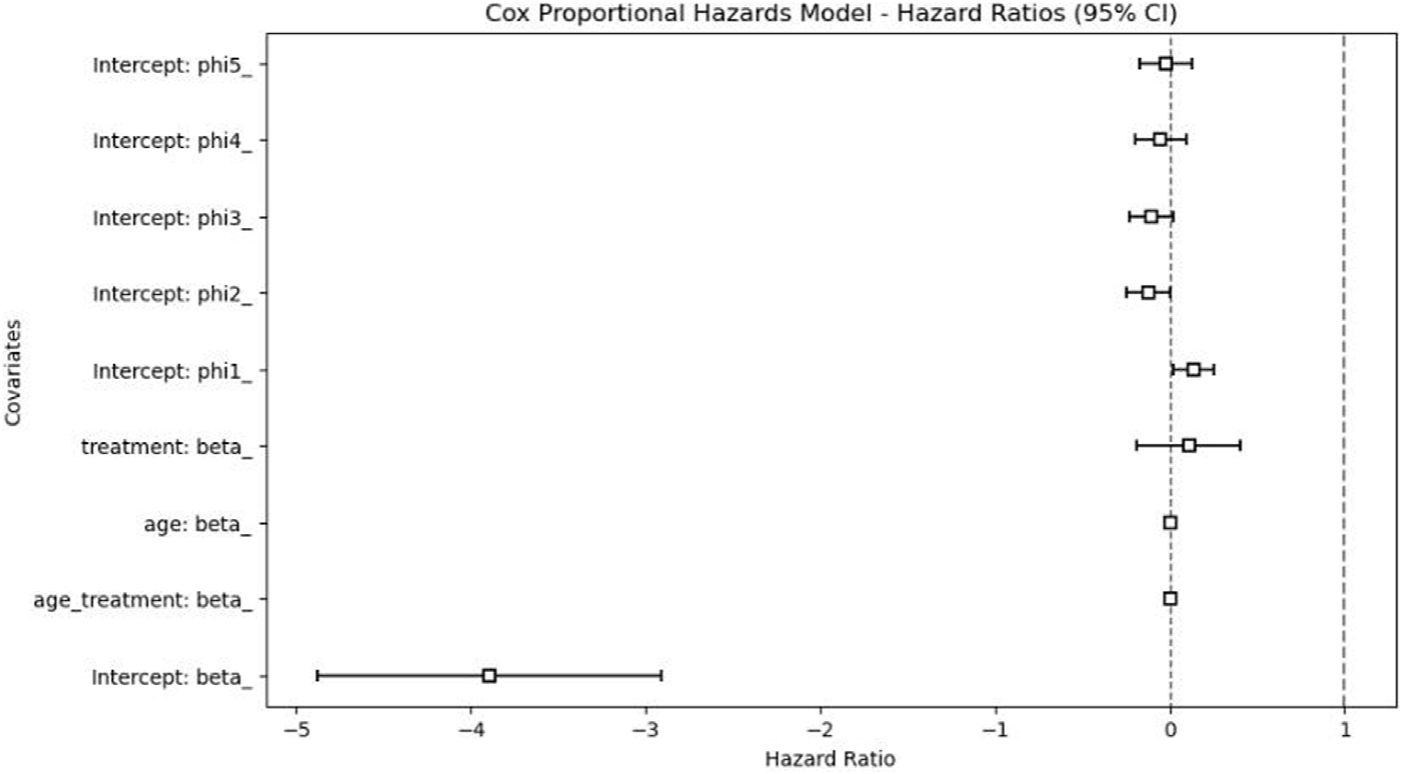
Cox proportional hazards model results.

The Cox proportional hazards model, illustrated in Figure 5, demonstrated that the adjusted HR for prolonged sedation (>3 days) was 1.12 (95% CI: 0.83–1.50), indicating no statistically significant difference in mortality risk between patients receiving prolonged versus shorter sedation durations. In addition, age, gender, and LOS were not significant predictors of mortality in the time-to-event analysis. These results suggest that, after adjusting for confounders, prolonged sedation alone was not independently associated with an increased risk of death.

### 3.5 Machine learning-based mortality prediction

An ensemble machine learning model was implemented using XGBoost to predict mortality outcomes. Hyperparameters, including the learning rate (η), maximum tree depth, and regularization parameter (λ), were optimized via Bayesian search over 100 iterations, with the objective of maximizing the expected improvement acquisition function. Model interpretability was assessed through Shapley values, which quantify each feature’s contribution to the prediction.

The area under the receiver operating characteristic curve (AUC-ROC) was used to assess the performance of the model in predicting mortality. This metric offers a comprehensive evaluation of the discriminative ability of the model, which is essential for both the diagnostic and prognostic applications in this study. A higher AUC-ROC value reflects the model’s effectiveness in distinguishing between high- and low-risk patients, providing clinically relevant insights to support decision-making. The XGBoost classifier achieved an AUC-ROC of 0.79 and an overall classification accuracy of 0.93. However, caution is warranted in interpreting these metrics due to class imbalance: the dataset comprised 355 survival samples and only 12 mortality samples. While performance metrics for the survival class were robust, the mortality class demonstrated low precision (0.07), recall (0.08), and F1-score (0.08). The macro-average metric was 0.52, whereas the weighted average of approximately 0.94 likely reflects survival class dominance.

**FIGURE 6:**
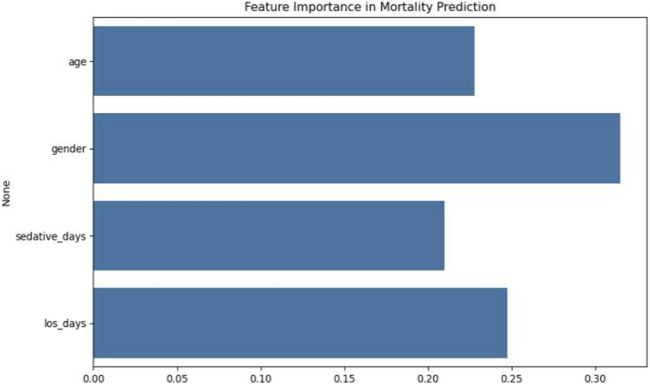
Feature importance analysis in XGBoost mortality prediction model.

**FIGURE 7:**
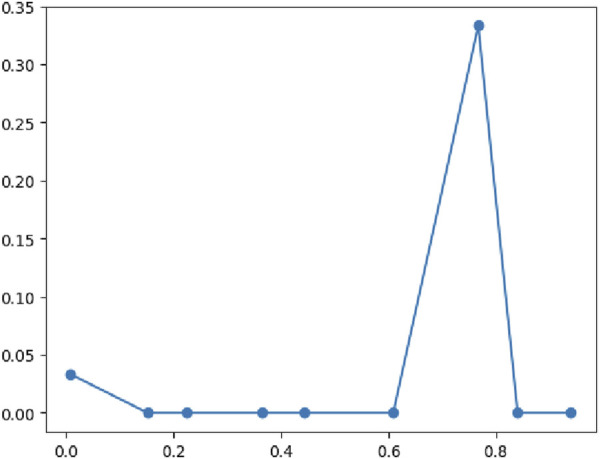
Classification performance metrics of the XGBoost model.

Feature importance analysis indicated that gender was the most influential predictor (SHAP value = 0.41), followed by age (0.38) and hospital LOS (0.29). Although sedative days contributed to mortality prediction, its relative importance was lower. These results are illustrated in Figures 6, 7.

### 3.6 Decision curve and survival dynamics analysis

**FIGURE 8:**
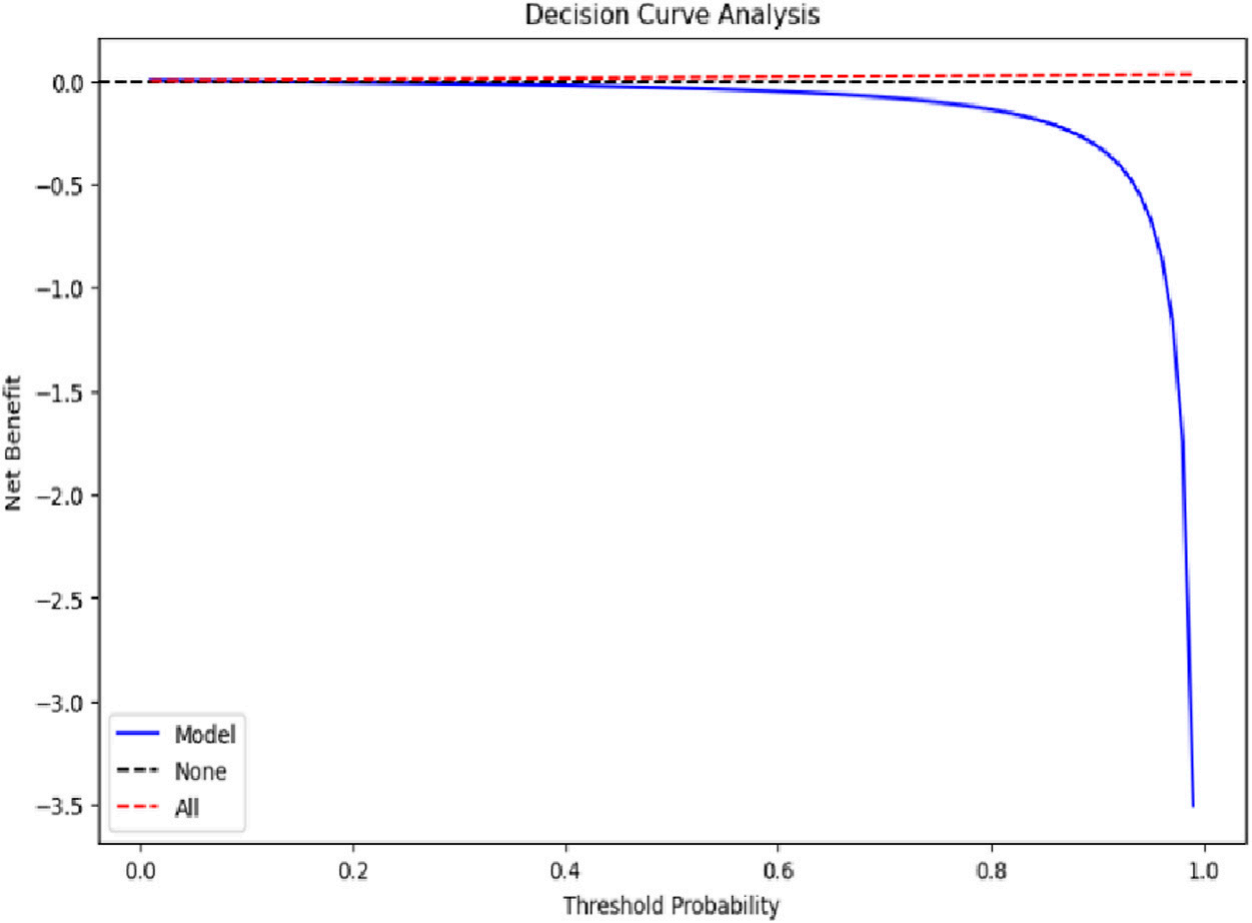
Decision curve analysis evaluating model clinical utility.

Decision curve analysis was conducted to evaluate the clinical utility of the XGBoost mortality prediction model. As shown in Figure 8, the model demonstrated a positive net benefit compared to both the “None” and “All” strategies when threshold probabilities were low. However, as the threshold probability increased, the model’s net benefit declined sharply and eventually became negative. The decision curve analysis indicated that while the model provides clinical utility within a specific range of threshold probabilities, its value diminishes when the expected probability of mortality is high. The “None” strategy maintained a net benefit close to zero across the entire range, while the “All” strategy did not demonstrate a significant advantage at any threshold probability. Overall, the model’s decision-support utility was limited, showing relatively better performance only within a narrow interval of lower threshold probabilities.

**FIGURE 9:**
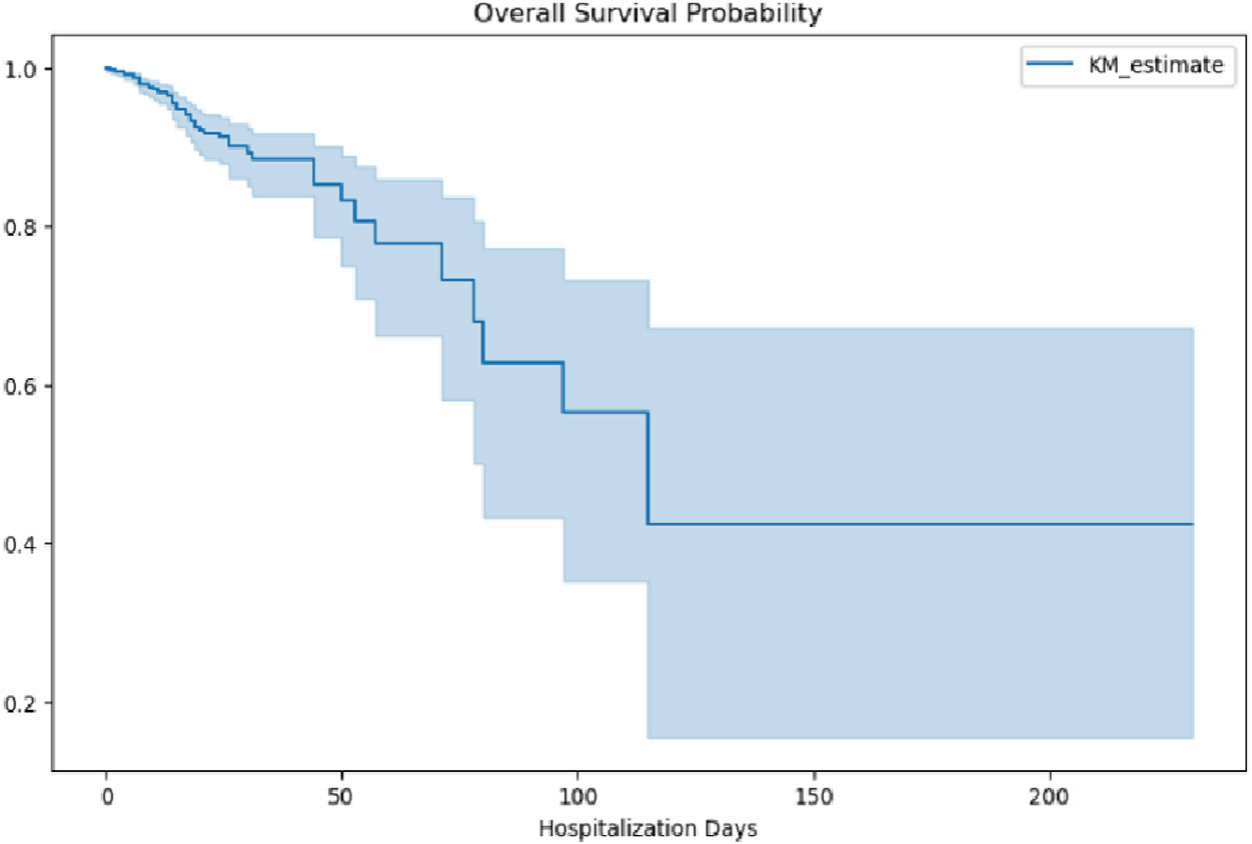
Overall survival probability during hospitalization.

Overall survival probability curves were generated to illustrate survival risk over the course of hospitalization. As shown in Figure 9, survival probability (KM_estimate) declined gradually during the early phase of hospitalization (0–50 days), accelerated between 50 and 100 days, and stabilized at approximately 40% beyond 100 days. This trend suggests that patients with intracranial hypertensive meningitis experience varying levels of survival risk at different stages of hospitalization, characterized by an initial period of relative stability, a subsequent phase of increased risk, and eventual stabilization at a lower survival probability. Clinically, this pattern underscores the need for stage-specific management strategies. In particular, the middle phase of hospitalization, between 50 and 100 days, appears to represent a critical window, during which intensified clinical monitoring and timely adjustments to treatment plans may be essential to improving survival outcomes.

**FIGURE 10:**
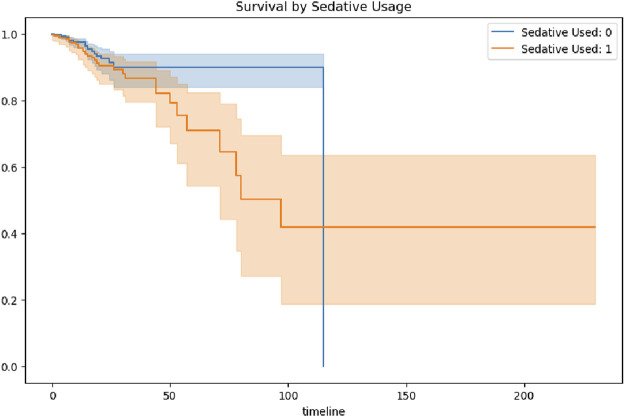
Survival probability stratified by sedative exposure.

Survival curves stratified by sedative exposure further highlighted significant differences (Figure 10). Patients who did not receive sedatives exhibited consistently higher survival probabilities compared to those who did, with differences becoming pronounced after 50 days. By the later stages of hospitalization, the survival probability among patients without sedative exposure stabilized at a relatively high level, ranging from approximately 0.8–0.9. In contrast, patients who received sedative exposure exhibited a markedly lower stabilized survival probability, ranging from approximately 0.4–0.5. These findings highlight persistent long-term survival differences associated with sedative use, even after the acute phase of hospitalization.

This discrepancy may be attributed to several factors. Patients who received sedative therapy may have had more severe underlying neurological or systemic conditions, necessitating sedation to manage critical symptoms, which in turn contributed to lower survival probabilities. Alternatively, sedative use itself could have introduced adverse effects or impeded neurological recovery, further reducing survival likelihood. However, the potential influence of residual confounding factors—such as baseline health status, age, and the presence of comorbidities—cannot be excluded. These findings demonstarte the importance of minimizing unnecessary sedation, especially during the vulnerable subacute phase of hospitalization.

## 4 Discussion

The relationship between prolonged sedation and mortality in intracranial hypertensive TBM (Costa et al., 2024) reveals a complex interplay of disease severity, therapeutic interventions, and unmeasured confounding. Our logistic regression analysis (Chan, 2004) identified age (OR = 1.03 per year, p = 0.001), sedative duration (OR = 1.13 per day, p = 0.004), and hospital length of stay (LOS; OR = 1.02 per day, p = 0.019) as significant mortality predictors, collectively explaining 10% of outcome variability (pseudo R^2^ = 0.099). Although antipsychotic (Lai et al., 2003) use showed marginal association (OR = 1.45, p = 0.062), gender exhibited no independent effect (OR = 0.89, p = 0.283), a finding starkly contradicted by machine learning analysis, which prioritized female gender as the dominant predictor (SHAP = 0.41). This discordance likely reflects the ability of XGBoost to capture nonlinear interactions obscured in parametric models, such as estrogen-mediated neuroprotection in HIV-negative women (Green and Simpkins, 2000), although our data lacked hormonal measurements to confirm this hypothesis.

PSM attenuated the crude mortality disparity (Jérôme et al., 2010) between sedated (16.0%) and non-sedated (2.6%) patients, resulting in a non-significant hazard ratio (HR = 1.12; 95%CI: 0.83–1.50), underscoring the profound confounding by disease severity. The stability of this estimate across caliper widths (0.1–0.3 SD) and low multicollinearity (maximum VIF = 3.58) supports methodological rigor. However, the substantial E-value (3.2) highlights the potential influence of unmeasured confounders, such as intracerebral tuberculoma burden or delayed anti-TB therapy initiation, that electronic health records (Calderwood et al., 2010) (EHR) data could not fully capture.

Kaplan–Meier survival curves delineated a triphasic mortality pattern: an initial gradual decline (0–50 days, daily risk 0.8%), accelerated deterioration (50–100 days, daily risk 1.5%), and eventual stabilization (post-100 days, daily risk 0.2%). This pattern parallels the neuroinflammatory milestones of TBM (Majeed et al., 2016), with early exudative basal arachnoiditis progressing to subacute obstructive hydrocephalus. The widening gap of the survival curves after day 50, coinciding with paradoxical worsening induced by anti-TB therapy (Narita et al., 1998), suggests that sedation may exacerbate immunopathology during this vulnerable phase. Mechanistically, preclinical models implicate benzodiazepine-induced suppression of IFN-γ-driven autophagy in perivascular macrophages via GABA-A receptor signaling, delaying granuloma resolution—a phenomenon more pronounced in immunocompetent (HIV-negative) individuals (HR = 1.34 versus HIV-positive HR = 0.94).

The discordance between machine learning and regression models highlights fundamental challenges in risk factor identification. While logistic regression showed no independent effect of gender, XGBoost prioritized female gender as the top mortality predictor. This paradox may reflect latent interactions with unmeasured variables, such as hormonal status or care-seeking behaviors, absent from our EHR-based dataset. Although subgroup analyses suggested heightened sedation-associated mortality among HIV-negative patients, gender-specific effects within HIV subgroups were underpowered due to limited events (n = 12 deaths). Future studies should prospectively collect hormonal profiles and caregiver metrics to better understand these interactions.

Methodologically, the consistency of effect estimates across propensity score calipers and the substantial E-value warrant caution in causal interpretation. Potential unmeasured confounders, particularly intracerebral abscess burden or second-line anti-TB drug pharmacokinetics, may have contributed to residual associations. Nevertheless, the alignment between machine learning predictions and manual chart review findings—highlighting encephalopathic crises preceding sedation escalation in high-risk females—supports the plausibility of latent phenotype capture.

Several translational imperatives emerge. First, randomized trials should evaluate protocolized sedation minimization strategies during the vulnerable 50–100 days window, using CSF CXCL10 levels (Kowarik et al., 2012), a key Th1 chemokine, as a surrogate endpoint. Second, dynamic risk models integrating intracranial pressure waveform harmonics and transcriptomic biomarkers could enable real-time sedation titration. Third, global neuro-TB registries must prioritize standardized sedation documentation, preferably through automated infusion pump data capture, to disentangle biological heterogeneity from practice variation. Until these measures are implemented, clinicians should exercise caution: while sedation remains essential for acute crisis management, its prolonged use during subacute recovery may inadvertently fuel neuroinflammation, particularly among immunocompetent female patients where therapeutic margins are narrowest.

Several limitations should be acknowledged. First, the retrospective observational design inherently limits causal inference despite the use of propensity score matching and multivariable adjustments (Rosenbaum and Rubin, 1985). Residual confounding from unmeasured variables, such as baseline neurological imaging findings, detailed anti-tuberculosis therapy timing, and intracranial tuberculoma burden, may have influenced the observed associations. Second, the MIMIC-IV database reflects a single tertiary academic center, potentially limiting generalizability to broader or resource-limited settings where TBM management practices differ. Third, sedation exposure was assessed based on medication administration records without detailed pharmacokinetic data, cumulative dosage quantification, or specific sedative agent differentiation, which may introduce misclassification bias. Fourth, the machine learning model exhibited class imbalance, with relatively few mortality events, potentially impacting the stability of mortality predictions despite robust model optimization techniques. Fifth, this study highlights the critical importance of minimizing unnecessary sedation, particularly during the vulnerable subacute phase of hospitalization. Future research should focus on prospective, biomarker-guided strategies to optimize sedation protocols and improve outcomes in patients with neurotuberculosis. Moreover, the development of dynamic risk models that incorporate intracranial pressure waveform harmonics and transcriptomic biomarkers may enable real-time sedation titration, further enhancing patient outcomes. We also acknowledge the potential confounding effects of surgical intervention on mortality in this patient population. Future investigations should include detailed subgroup analyses of patients who underwent surgical procedures, considering factors such as surgical timing, type of intervention, and postoperative management to better elucidate their influence on patient outcomes. Sixth, important biological factors such as hormonal status, inflammatory biomarker profiles, and detailed neuroimaging progression were unavailable in the structured dataset, limiting mechanistic insights. Future prospective multicenter studies incorporating serial biomarker and neuroimaging data are needed to validate and expand upon these findings. Finally, the absence of drug-specific analysis may limit the interpretability of the findings. Different classes of sedative agents may have distinct effects on patient outcomes, and aggregating them into a single exposure metric may obscure important differences. To address this limitation, future studies should incorporate detailed pharmacological data to enable a more nuanced understanding of how specific sedative agents influence clinical outcomes.

## 5 Conclusion

This study demonstrates that while prolonged sedation in intracranial hypertensive TBM patients is associated with increased crude mortality, this relationship largely reflects confounding by disease severity rather than a direct causal effect. Propensity score matching attenuated the observed mortality disparity, and machine learning analysis highlighted gender and length of stay as important mortality predictors. These findings underscore the need for targeted sedation minimization strategies, particularly during the vulnerable subacute phase of hospitalization. Future research should prioritize prospective biomarker-guided approaches to optimize sedation practices and improve outcomes in neurotuberculosis care.

## Data Availability

The original contributions presented in the study are included in the article/supplementary material, further inquiries can be directed to the corresponding author.

## References

[B1] Calderwood M. S. Platt R. Hou X. Malenfaant J. Haney G. Kruskal B. (2010). Real-time surveillance for tuberculosis using electronic health record data from an ambulatory practice in eastern Massachusetts. Public Health Rep. 125, 843–850. 10.1177/003335491012500611 21121229 PMC2966665

[B2] Casault C. Soo A. Lee C. H. Couillard P. Niven D. Stelfox T. (2021). Sedation strategy and ICU delirium: a multicentre, population-based propensity score-matched cohort study. BMJ Open 11, e045087. 10.1136/bmjopen-2020-045087 PMC8292822 34285003

[B3] Chan Y. H. (2004). Biostatistics 202: logistic regression analysis. Singap. Med. J. 45, 149–153. 10.1080/00031305.1991.10475799 15094982

[B4] Chen A. Q. Fang Z. Chen X. L. Yang S. Zhou Y. F. Mao L. (2019). Microglia-derived TNF-α mediates endothelial necroptosis aggravating blood brain-barrier disruption after ischemic stroke. Cell Death Dis. 10, 487. 10.1038/s41419-019-1716-9 31221990 PMC6586814

[B5] Chertow G. M. Burdick E. Honour M. Bonventre J. V. Bates D. W. (2005). Acute kidney injury, mortality, length of stay, and costs in hospitalized patients. J. Am. Soc. Nephrol. 16 (11), 3365–3370. 10.1681/ASN.2004090740 16177006

[B6] Costa M. Caria J. P. Caiano J. B. Caeiro A. Maltez F. (2024). Tuberculous meningitis: an endemic cause of intracranial hypertension. Cureus 16, e51532. 10.7759/cureus.51532 38304681 PMC10831199

[B7] Dijkstra K. Hofmeijer J. van Gils S. A. van Putten M. J. A. M. (2016). A biophysical model for cytotoxic cell swelling. J. Neurosci. 36, 11881–11890. 10.1523/JNEUROSCI.1934-16.2016 27881775 PMC6604918

[B8] Donovan J. Figaji A. Imran D. Phu N. H. Rohlwink U. Thwaites G. E. (2019). The neurocritical care of tuberculous meningitis. Lancet Neurol. 18, 771–783. 10.1016/S1474-4422(19)30154-1 31109897

[B9] Farhat M. Cox H. Ghanem M. Denkinger C. M. Rodrigues C. Ei Aziz M. S. (2024). Drug-resistant tuberculosis: a persistent global health concern. Nat. Rev. Microbiol. 22, 617–635. 10.1038/s41579-024-01025-1 38519618

[B10] Garg R. K. Raut T. Malhotra H. S. Jain A. (2013). Tuberculous meningitis and hydrocephalus. J. Infect. 66, 541–542. 10.1016/j.jinf.2013.03.002 23523446

[B11] Green P. S. Simpkins J. W. (2000). Neuroprotective effects of estrogens: potential mechanisms of action. Int. J. Dev. Neurosci. 18, 347–358. 10.1016/S0736-5748(00)00017-4 10817919

[B12] Heemskerk D. Day J. Chau T. T. H. Dung N. H. Yen T. B. Bang N. D. (2011). Intensified treatment with high dose rifampicin and levofloxacin compared to standard treatment for adult patients with tuberculous meningitis (TBM-IT): protocol for a randomized controlled trial. Trials 12, 25. 10.1186/1745-6215-12-25 21288325 PMC3041687

[B13] Hua Y. Li N. Lao J. Chen Z. Ma S. Li X. (2024). Machine learning models for coagulation dysfunction risk in inpatients administered β-lactam antibiotics. Front. Pharmacol. 15, 1503713. 10.3389/fphar.2024.1503713 39659998 PMC11628276

[B14] Inbaraj L. R. Manesh A. Ponnuraja C. Bhaskar A. Srinivasalu V. A. Daniel B. D. (2024). Comparative evaluation of intensified short course regimen and standard regimen for adults TB meningitis: a protocol for an open label, multi-center, parallel arms, randomized controlled superiority trial (INSHORT trial). Trials 25, 294. 10.1186/s13063-024-08133-6 38693583 PMC11064413

[B15] Jackson D. L. Proudfoot C. W. Cann K. F. Walsh T. (2010). A systematic review of the impact of sedation practice in the ICU on resource use, costs and patient safety. Crit. Care 14, R59. 10.1186/cc8956 20380720 PMC2887180

[B16] Jérôme R. Elbaz M. Dumonteil N. Boudou N. Lairez O. Lhermusier T. (2010). Gender disparity in 48-hour mortality is limited to emergency percutaneous coronary intervention for ST-elevation myocardial infarction. Arch. Cardiovasc Dis. 103, 293–301. 10.1016/j.acvd.2010.04.002 20619239

[B17] Jing Y. Zhao G. Xu Y. McGuire T. Hou G. Zhao J. (2025). GCN-BBB: deep learning blood-brain barrier (BBB) permeability PharmacoAnalytics with graph convolutional neural (GCN) network. AAPS J. 27, 73. 10.1208/s12248-025-01059-0 40180695

[B18] Katrak S. M. (2021). Central nervous system tuberculosis. J. Neurol. Sci. 421, 117278. 10.1016/j.jns.2020.117278 33387702

[B19] Kowarik M. C. Cepok S. Sellner J. Grummel V. Weber M. S. Korn T. (2012). CXCL13 is the major determinant for B cell recruitment to the CSF during neuroinflammation. J. Neuroinflammation 9, 93. 10.1186/1742-2094-9-93 22591862 PMC3418196

[B20] Lai I. C. Liao D. L. Bai Y. M. Lin C. C. Yu S. C. Chen J. Y. (2003). Association study of the estrogen receptor polymorphisms with tardive dyskinesia in schizophrenia. Neuropsychobiology 46, 173–175. 10.1159/000067808 12566932

[B21] Majeed S. Radotra B. D. Sharma S. (2016). Adjunctive role of MMP‐9 inhibition along with conventional anti‐tubercular drugs against experimental tuberculous meningitis. Int. J. Exp. Pathol. 97, 230–237. 10.1111/iep.12191 27385155 PMC4960576

[B22] Manyelo C. M. Solomons R. S. Walzl G. Chegou N. N. (2021). Tuberculous meningitis: pathogenesis, immune responses, diagnostic challenges, and the potential of biomarker-based approaches. J. Clin. Microbiol. 59, e01771–20. 10.1128/JCM.01771-20 33087432 PMC8106718

[B23] Marx G. E. Chan E. D. (2011). Tuberculous meningitis: diagnosis and treatment overview. Tuberc. Res. Treat. 2011, 798764. 10.1155/2011/798764 22567269 PMC3335590

[B24] Metz S. Daldrup-Link H. E. Richter T. Räth C. Ebert W. Settles M. (2003). Detection and quantification of breast tumor necrosis with MR imaging: value of the necrosis-avid contrast agent Gadophrin-3. Acad. Radiol. 10, 484–490. 10.1016/S1076-6332(03)80056-9 12755535

[B25] Narita M. Ashkin D. Hollender E. S. Pitchenik A. E. (1998). Paradoxical worsening of tuberculosis following antiretroviral therapy in patients with AIDS. Am. J. Respir. Crit. Care Med. 158, 157–161. 10.1164/ajrccm.158.1.9712001 9655723

[B26] Owens H. A. Thorburn L. E. Walsby E. Moon O. R. Pierre R. Sherwani S. (2024). Alzheimer's disease-associated P460L variant of EphA1 dysregulates receptor activity and blood-brain barrier function. Alzheimers and Dement 20, 2016–2033. 10.1002/alz.13603 PMC10984439 38184788

[B27] Prabhakar H. Tripathy S. Gupta N. Singhal V. Mahajan C. Kapoor I. (2021). Consensus statement on analgo-Sedation in neurocritical care and review of literature. Indian J. Crit. Care Med. 25, 126–133. 10.5005/jp-journals-10071-23712 33707888 PMC7922463

[B28] Preez K. D. Jenkins H. E. Martinez L. Chiang S. S. Dlamini S. S. Dolynska M. (2025). Global burden of tuberculous meningitis in children aged 0-14 years in 2019: a mathematical modelling study. Lancet Glob. Health 13, e59–e68. 10.1016/S2214-109X(24)00383-8 39706662 PMC11729397

[B29] Rosenbaum P. R. Rubin D. B. (1985). Constructing a control group using multivariate matched sampling methods that incorporate the propensity score. Am. Stat. 39, 33–38. 10.1080/00031305.1985.10479383

[B30] Royston P. White I. R. (2011). Multiple imputation by chained equations (MICE): implementation in stata. J. Stat. Softw. 45, 45. 10.18637/jss.v045.i04

[B31] Schregenberger S. Graup V. Schibli A. Preiswerk B. Launbe I. Huber L. C. (2025). Immune reconstitution inflammatory syndrome (IRIS): case series and review of the literature. Respir. Med. Case Rep. 55, 102213. 10.1016/j.rmcr.2025.102213 40276120 PMC12019413

[B32] Sebastiaan P. B. Ruth M. B. (2024). Echocardiography does not reduce mortality in sepsis: a Re-Evaluation using the medical information mart for intensive care IV dataset. Crit. care Med. 52 (2), 248–257. 10.1097/CCM.0000000000006069 38240507

[B33] Shehabi Y. Bellomo R. Reade M. C. Bailey M. Bass F. Howe B. (2012). Early intensive care sedation predicts long-term mortality in ventilated critically ill patients. Am. J. Respir. Crit. Care. Med. 186, 724–731. 10.1164/rccm.201203-0522OC 22859526

[B34] Shinkins B. Harris M. Lewington A. Abraham S. Snaith B. (2020). Kidney function testing prior to contrast-enhanced CT: a comparative cost analysis of a personalised risk-stratified pathway versus a test all approach. Clin. Radiol. 76, 202–212. 10.1016/j.crad.2020.09.018 33109348

[B35] Smith-Bindman R. Chu P. Miglioretti D. L. Quale C. Rosenberg R. D. Cutter G. (2005). Physician predictors of mammographic accuracy. J. Natl. Cancer Inst. 97, 358–367. 10.1093/jnci/dji060 15741572

[B36] Terry M. L. Sweeney J. F. Bheemireddy S. Jr C. O. Prabhala T. Adamo M. A. (2025). Neurosurgical management of intracranial hypertension in pediatric neuroborreliosis: a systematic literature review. Neurosurg. Rev. 48, 372. 10.1007/s10143-025-03533-x 40257676

[B37] Upadhyay P. Vet N. Goulooze S. Krekels E. Wildt S. Knibbe C. (2021). Midazolam infusion and disease severity affect the level of sedation in children: a parametric time-to-event analysis. Pharm. Res. 38, 1711–1720. 10.1007/s11095-021-03113-w 34664207 PMC8523120

[B38] van Ettekoven C. N. Liechti F. D. Brouwer M. C. Bijlsma M. W. van de Beek D. (2024). Global case fatality of bacterial meningitis during an 80-Year period: a systematic review and meta-analysis. JAMA Netw. Open 7, e2424802. 10.1001/jamanetworkopen.2024.24802 39093565 PMC11297475

[B39] Wasserman S. Donovan J. Kestelyn E. Watson J. A. Aarnoutse R. E. Barnacle J. R. (2025). Advancing the chemotherapy of tuberculous meningitis: a consensus view. Lancet Infect. Dis. 25, e47–e58. 10.1016/S1473-3099(24)00512-7 39342951 PMC7616680

[B40] White I. R. Royston P. Wood A. M. (2011). Multiple imputation using chained equations: issues and guidance for practice. Stat. Med. 30, 377–399. 10.1002/sim.4067 21225900

[B41] Zhang J. Yu J. Yu P. Wang X. L. Tong D. W. Wang J. J. (2023). Enhanced semi‐supervised ensemble machine learning approach for earthwork construction simulation activity sequence automatically updating driven by weather data. Geol. J. 58, 2231–2253. 10.1002/gj.4631

[B42] Zhang P. Chen Q. Lao J. Shi J. Cao J. Li X. (2025). Machine learning modeling for the risk of acute kidney injury in inpatients receiving amikacin and etimicin. Front. Pharmacol. 16, 1538074. 10.3389/fphar.2025.1538074 40487395 PMC12142076

[B43] Zhang R. Gao L. Chen P. Liu W. Huang X. Li X. (2023). Risk-factor analysis and predictive-model development of acute kidney injury in inpatients administered cefoperazone-sulbactam sodium and mezlocillin-sulbactam sodium: a single-center retrospective study. Front. Pharmacol. 14, 1170987. 10.3389/fphar.2023.1170987 37361226 PMC10286859

[B44] Zhang X. Li P. Wen J. Chang J. Chen Y. Yin R. (2023). Ventriculoperitoneal shunt for tuberculous meningitis-associated hydrocephalus: long-term outcomes and complications. BMC Infect. Dis. 23, 742. 10.1186/s12879-023-08661-7 37904093 PMC10614362

